# Expression and Purification of Functional Human Mu Opioid Receptor from *E.coli*

**DOI:** 10.1371/journal.pone.0056500

**Published:** 2013-02-21

**Authors:** Yanbin Ma, Jan Kubicek, Jörg Labahn

**Affiliations:** 1 Institute of Complex Systems (ICS-6), Research Center Jülich, Jülich, Germany; 2 QIAGEN GmbH, Hilden, Germany; The University of Manchester, United Kingdom

## Abstract

N-terminally his-tagged *human mu opioid receptor*, a G protein-coupled receptor was produced in *E.coli* employing synthetic codon-usage optimized constructs. The receptor was expressed in inclusion bodies and membrane-inserted in different *E.coli* strains. By optimizing the expression conditions the expression level for the membrane-integrated receptor was raised to 0.3–0.5 mg per liter of culture. Milligram quantities of receptor could be enriched by affinity chromatography from IPTG induced cultures grown at 18°C. By size exclusion chromatography the protein fraction with the fraction of alpha-helical secondary structure expected for a 7-TM receptor was isolated, by CD-spectroscopy an alpha-helical content of ca. 45% was found for protein solubilised in the detergent Fos-12. Receptor in Fos-12 micelles was shown to bind endomorphin-1 with a K_D_ of 61 nM. A final yield of 0.17 mg functional protein per liter of culture was obtained.

## Introduction

G protein-coupled receptors (GPCRs) are the largest family of integral membrane proteins which account for up to 50% of all drug targets including cardiovascular and gastrointestinal diseases, central nervous system and immune disorders, cancer and pain [1], [2], [3], [4], [5].

Opioid receptors have been classified into three different types, μ, δ, κ [6]. The μ type human mu-opioid receptor OPRM is activated by endogenous opioid peptides such as beta-endorphins and exogenous alkaloids such as morphine. OPRM plays very important roles in regulating several physiological processes such as pain, stress, and emotions [7], [8]. Although GPCRs represents major pharmaceutical targets, only few structural data on GPCRs have been obtained. This is mainly due to the hydrophobicity of these proteins, very low natural abundance, difficulties in overexpression and purification and low stability after extraction from the membrane environment [9]. Recently the crystal structure of human OPRM with T4 lysozyme inserted in 3^rd^ intracellular loop was determined [10].

Many studies have focused on expression and purification of functional GPCRs to obtain the required material for biological analysis and crystallization [11], [12], [13]. To solve the problem of yield, in addition to modifications in the gene sequence, several expression strategies carried out with bacterial [14], [15], yeast [16], [17], [18] and higher eukaryotic host systems [19], [20], [21]. These experiments showed that the expression levels of functional GPCRs could be improved by optimization of the expression conditions: GPCRs were found to be often (i) toxic to *E. coli*, (ii) subject to degradation or (iii) inclusion body formation [22], (iv) difficult to solubilise.

Expression of GPCRs in *E.coli* has shown very low yields [23]. It was reported that Human μ, δ, κ opioid receptors were successfully expressed in *E.coli* when fused to periplasmic maltose-binding protein (MBP). However, an average of only 30 correctly folded receptor molecules per cell for the three subtypes were found [14]. Milligram amounts of the full length mu-opioid receptor (alone and in fusion with enhanced green fluorescent protein, EGFP) have been obtained as inclusion bodies in *Pichia pastoris* [8]. μ-opioid receptor fused to yellow fluorescent protein was expressed in insect cells with a reproducible yield of only 50 µg functional receptor/liter of insect culture [24].

Expression in *E.coli* allows generally for easy scale up and avoids problems with posttranslational modifications and GPCR hetero-oligomerization with GPCRs of the host cells [25]. However, overexpression of membrane proteins in membrane-integrated form in *E.coli* is usually toxic to the organism and thus leads to reduction in yields [26], presumably due to the limitation of the *E.coli* membrane space and different membrane translocation system. It has been reported that several functional GPCRs were successfully expressed via *E.coli* [12], [14], [23], [27] or *E.coli* cell-free system [11], [28], [29].

Here, we investigated the possibility to obtain by heterologous expression in *E.coli* functional human mu-opioid receptor, which is modified only by a removable his-tag to facilitate enrichment and identification upon purification, but does not contain any stabilizing modifications like insertion of T4 lysozyme [10] that may affect the expected structural changes of the receptor when performing the signaling function.

## Results

### Expression of a Membrane-inserted OPRM in *E.coli*

Various *E.coli* strains (RP, RIL, C41, and C43) were screened for expression of the target protein. The parameters temperature (18°C and 37°C), induction time, expression medium (DYT and TB) and induction method (0.2–0.8 mM IPTG or autoinduction) were varied to optimize the expression level.

**Figure 1:**
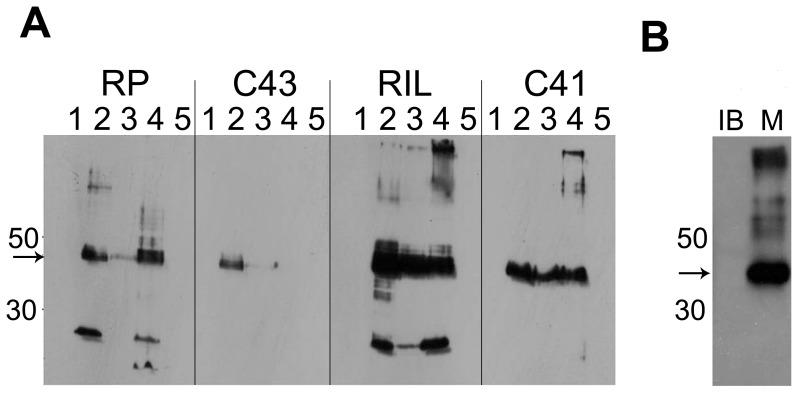
Expression of the N-terminally his-tagged OPRM protein. Western blot on His-tag. A, Expression by autoinduction at 37°C in different E.coli strains (RP, RIL, C41, and C43). Lane 1 - uninduced, lane 2–Inclusion body fraction (induced 4 h), lane 3–Membrane fraction (induced 4 h), lane 4–Inclusion body fraction (induced 20 h), lane 5–Membrane fraction (induced 20 h). B, Optimised expression of OPRM using C43 cells, TB medium with 0.4 mM IPTG at 18°C. Western blot showed inclusion body (IB) and membrane fractions (M) of OPRM.

At high temperature (37°C), the N-terminal his-tagged OPRM was found to be produced both in inclusion bodies and in membrane-inserted form (Figure 1A): for C41 cells only a low expression level was observed, most of the target protein was found in the inclusion bodies. For other cells at higher expression levels OPRM was increasingly found in form of inclusion bodies or even degraded as seen for the case of expression in RIL cells, where 30–50% of OPRM was degraded into a large N-terminal fragment (ca. 18 k Da).

**Figure 2:**
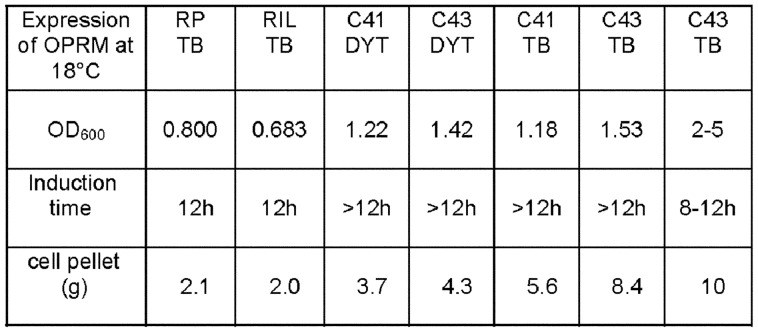
Growth conditions of OPRM in different E.coli strains. Expression of OPRM was induced by IPTG. Cell culture density (OD_600_) and weight of cell pellet (g) after different induction times with two different media (TB and DYT) was measured. Cell pellet (g) was obtained from 1 liter of culture medium.

Upon induction with IPTG at 37°C severe foam formation with loss of cell density was observed. Typically the culture decayed within 3 hours after induction. Thus the expression of the OPRM was found to be toxic. Very slow growth of the culture was observed for induction at 18°C. These results indicated a proper harvesting time and induction period should be optimized even for expression at 18°C. Extended induction time (>12 h) led to low cell density (OD600<2), whereas a proper induction time of less than 10 h was optimal to maximize cell yield (Final OD_600_ = 2–5, cell pellet >8 g/l) in all cases. With the richer medium TB more cells could be harvested (Figure 2).

**Figure 3:**
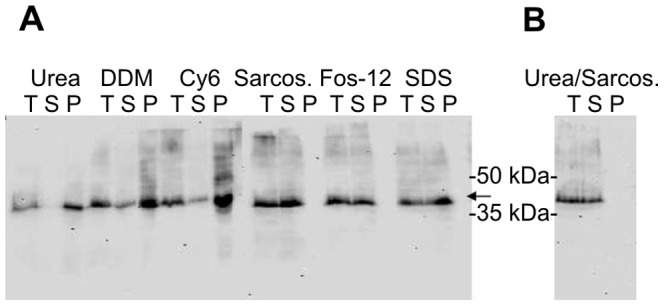
Solubilisation of OPRM with detergents. A, solubilisation with urea or detergents. B, solubilisation with urea and laurylsarcosine. T -total membrane fraction, S -solubilised membrane fraction, P -pellet after solubilisation.

The optimized IPTG concentration (0.4 mM) was found to effectively induce the expression of OPRM, while increasing IPTG concentration led to degradation of the protein or to the formation of inclusion bodies. With the conditions of 0.4 mM IPTG at 18°C for 8–12 h in C43 almost no inclusion bodies were produced within C43. OPRM was obtained in the membrane fraction (Figure 1B). The optimal expression level of OPRM was determined to be 0.3–0.5 mg/liter of culture by complete solubilisation of the protein in the membrane fraction under denaturating conditions with 6 M urea and 0.8% laurylsarcosine (Figure 3B) and subsequent western blot.

Remarkably, no appreciable expression of OPRM with a C-terminal his-tag was observed under any of the tested conditions (data not shown).

### OPRM Solubilisation

Solubilisation of membrane protein from the membrane is one of the crucial steps of purification, which is routinely achieved by optimizing the detergent to minimize denaturation during solubilisation. Therefore a variety of detergents were used to extract OPRM from *E.coli* membrane and as controls: Zwitterionic detergents (1% (w/v) LDAO, 1% (w/v) Fos-12), nonionic detergents (1% (w/v) DDM, 1% (w/v) Cy6) and anionic detergent (1% (w/v) SDS, 0.8% (w/v) laurylsarcosine with/without 6 M urea). The detergents for the isolation of folded protein were chosen to cover the typical range of micelle aggregation numbers (10–133) and a reduced range of hydrophile-lipophile balances (HLB: 5.3 to 14.2) [30]. The more hydrophilic detergents with HLB>14.2 were excluded because complete solubilisation of the target protein was aimed for.

Urea without detergent showed very poor solubilisation efficiency. The receptor remained in the pellet upon solubilisation, indicating the receptor was located in the membrane. Solubilisation using mild detergents turned out to be only moderately successful. Extraction of OPRM with SDS, laurylsarcosine alone, or 6 M urea with 0.8% (w/v) laurylsarcosine proved to be most efficient (Figure 3A and B). The detergent Fos-12 was outstanding in solubilisation of the receptor. No residual receptor was found in the pellet after solubilisation.

### Isolation of OPRM

Purification of OPRM was carried out with several purification strategies such as affinity chromatography, ionic exchange chromatography and size exclusion chromatography. Ionic exchange chromatography was found to be of limited value in purification of the membrane protein especially when solubilised with an ionic or zwitterionic detergent.

**Figure 4:**
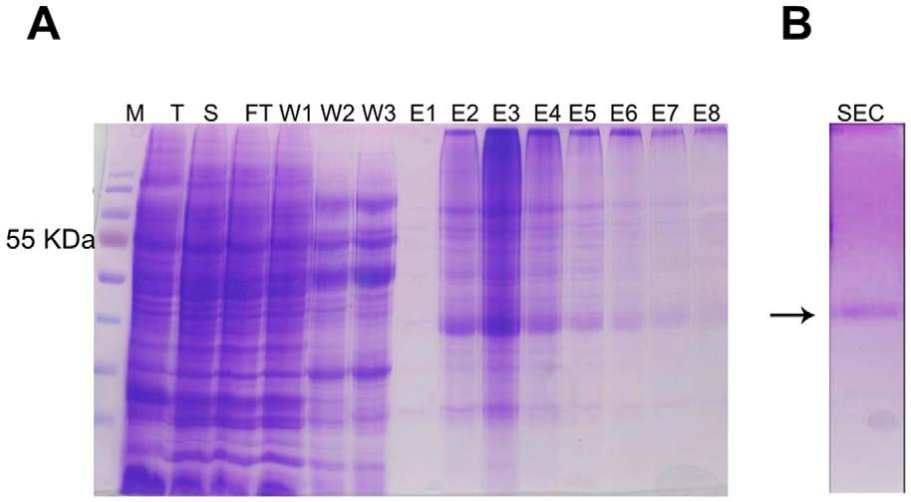
Purification of OPRM from C43 in Fos-12. A, Purification of OPRM solubilised with Fos-12 by Ni-NTA. T -total membrane fraction, S -solubilised membrane fraction, FT -flowthrough. W – wash fractions (25 mM imidazole), E – elution fractions (300 mM imidazole). The arrow denotes the monomeric OPRM. B, SEC -purified OPRM after size exclusion chromatography.

OPRM extracted from membrane was purified through metal chelate affinity chromatography (NI-NTA) two times, followed by size exclusion (Superdex 200) chromatography. In the first purification step the majority of OPRM can be captured by Ni-NTA (Figure 4A). A second Ni-NTA chromatography of the diluted sample improves the purity to ca. 85%.

**Figure 5:**
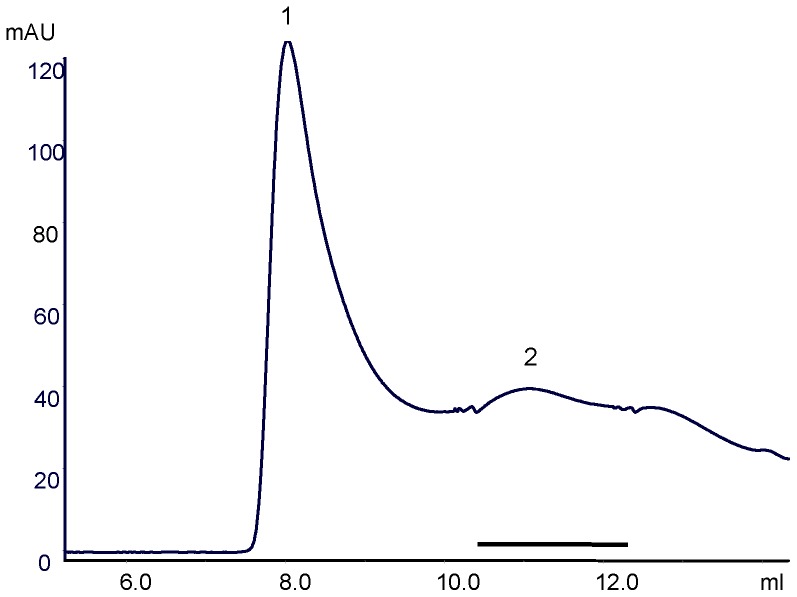
Size exclusion chromatography of OPRM in Fos-12. Purification of OPRM was performed in analytical grade Superdex 200 HR 10/30 size exclusion chromatography. Peak 1 identifies the aggregation of OPRM. The underlined Peak 2 shows the monomeric form of OPRM.

Residual impurities and aggregated material were removed by (SEC) size exclusion chromatography (Figure 4B). It was also used to assess the state of aggregation of OPRM (Figure 5): Peak 1 (Superdex 200 HR 10/30, GE Healthcare in 0.1% (w/v) Fos-12) shows aggregated protein. It was regarded to be caused by the instability of the protein in detergent, respectively the presence of misfolded and unfolded protein. Thus a final yield of 0.17 mg/liter of culture was obtained by Ni-NTA and size exclusion chromatography (Figure 4B). The elution profile of the receptor shows a peak with an apparent molecular weight of the Fos-12/receptor complex of ca. 158 kDa (underlined in Figure 5). The expected molecular weight of the Fos-12/receptor complex is ca. 65 kDa (Mw of OPRM 46 kD, and Mw of Fos-12 micelle (in H_2_O) ∼19 kD). It appears that the apparent molecular weight for this Fos-12/receptor complex does not agree with the expected molecular weight of the monomeric detergent-receptor complex. The difference between the predicted and the observed Mw might be due to non-ideal behavior of the detergent/receptor complex in the size exclusion column or dimerisation.

### Confirmation of Full Length of OPRM

OPRM, western blot positive for the N-terminal his-tag, was found at a position of around 38 kDa on 12% SDS-PAGE (Figure 4), though the expected Mw is 46 kDa. Several integral membrane proteins including several GPCRs were found to migrate anomalously smaller than expected on SDS–PAGE due to their hydrophobicity and compact structure [30]. Nevertheless, the presence of the full-length protein had to be confirmed.

**Figure 6:**
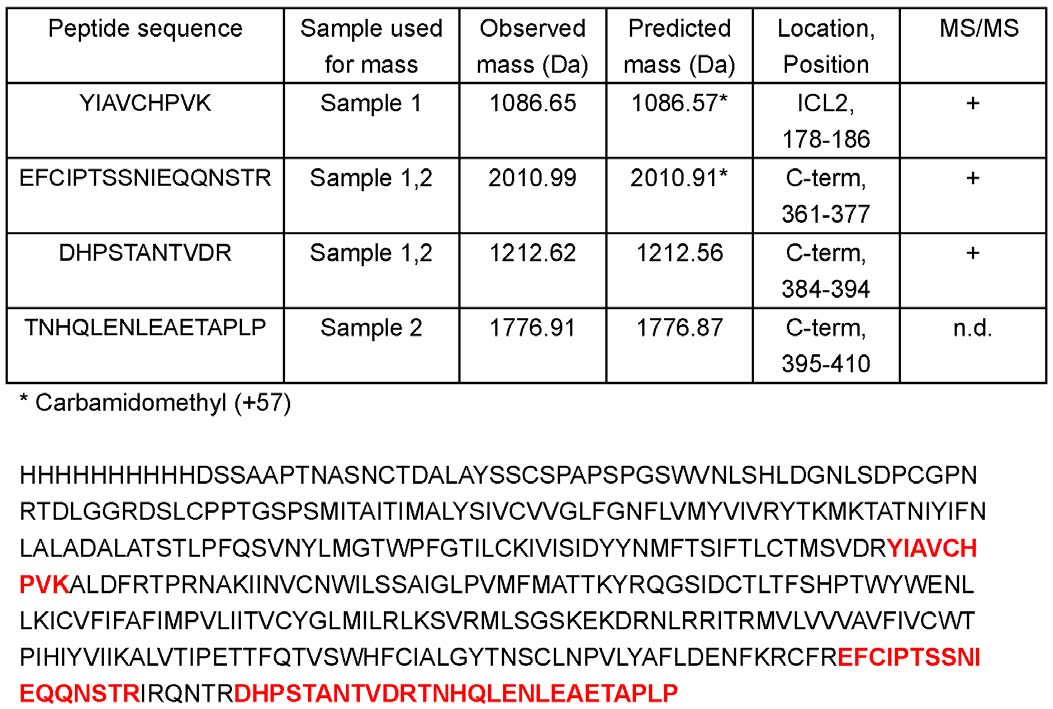
Mass spectrometry of OPRM. Sequence coverage of trypsin digested peptide fragments identified. MS/MS spectrum of an identified peptide fragment EFCIPTSSNIEQQNSTR and OPRM sequence with identified fragments in red.

The protein was extracted from SDS-PAGE, digested with trypsin and treated with iodoacetamide and DTT for analysis by mass spectrometry. Only after treatment with DTT and iodoacetamide before digestion with trypsin peptide matches were found (Figure 6A). Four matches were further analyzed by MS/MS analysis. These peptides were derived from cytoplasmic and intracellular loops connecting transmembrane domains, but not from the N-terminal domain that does not contain a trypsin cleavage site. A total of 13% sequence coverage was obtained (Figure 6B). As the C-terminal peptide was also found, the band with apparent Mw of 38 kDa in SDS-PAGE corresponded to the full length of the 46 kDa protein.

### Confirmation of 7-TM Alpha-helical Secondary Structure

**Figure 7:**
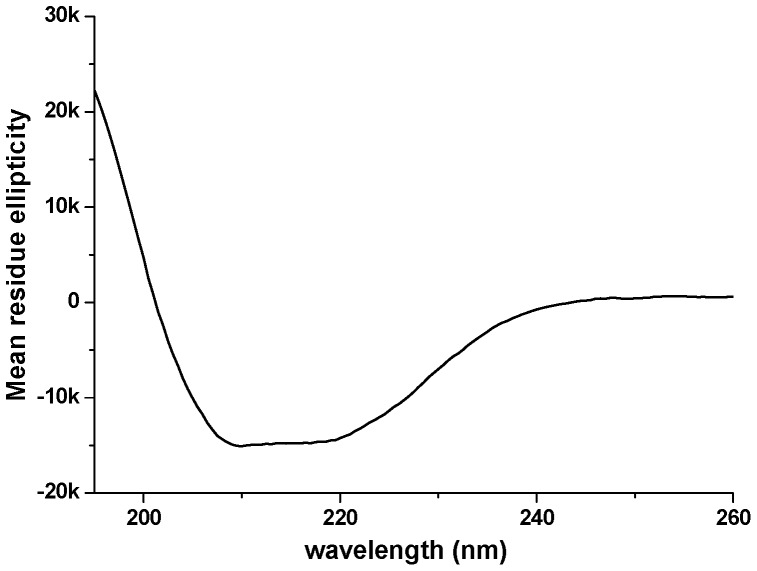
Secondary structural analysis of purified OPRM protein. The Circular dichroism spectrum of OPRM at 25°C. Mean residue ellipticity [θ] in degrees×cm^2^×dmol^−1^.

A first characterization of OPRM receptor natively purified from bacterial membrane was carried out by circular dichroism. The secondary structure of the purified OPRM after gel filtration was determined by CD-data from the far-UV spectrum in the 200–250 nm range (Figure 7) by K2D deconvolution. The folded protein was characterized to have a secondary structure of 46±5% alpha-helix. The prediction for the receptor, based on free web SOPMA calculations, is 43% alpha-helix. The agreement of observation and expectation is evidence for a correctly folded receptor having seven helical transmembrane domains. Material isolated in Peak 1 (Figure 5) was found to have an alpha-helical content corresponding to 5–6 TM-helices (data not shown).

### Confirmation of Receptor Function by Agonist Binding

The functionality of the isolated OPRM was probed by measuring the binding of the natural ligand endomorphine-1 to OPRM by plasmon surface resonance. Initially about 8000 RUs of OPRM (MW 46 kDa) were bound to the Ni-NTA chip. After extensively washing with buffer ca. 4000 RU remained. These results illustrated that for membrane proteins high initial responses may be observed because of unspecific binding or aggregation. The addition of reducing agent (1 mM TCEP) to the loading buffer did not change the binding of OPRM.

**Figure 8:**
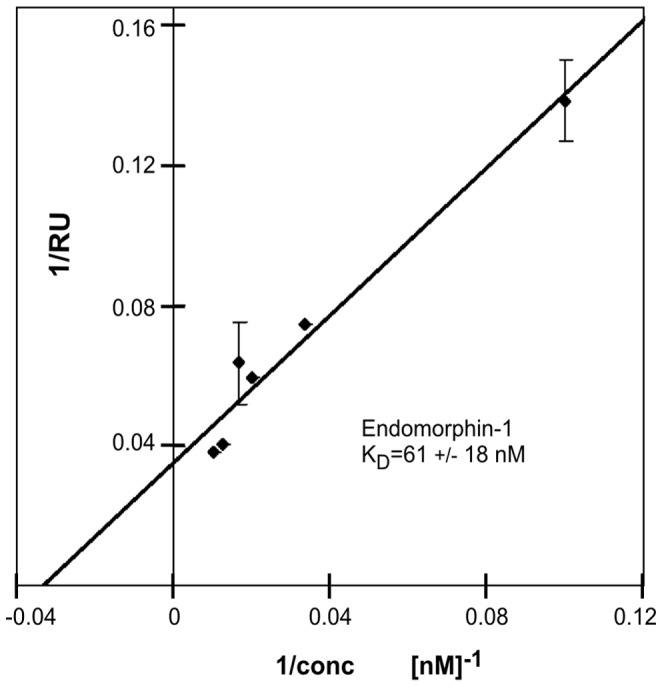
Interaction of OPRM with Endomorphin-1 by Surface Plasmon Resonance (SPR). SPR shows the apparent association increases in RU response with the addition of EM-1 at 25°C. The binding constant (K_D_) of EM-1 to OPRM was obtained from (Rmax-R)*C/R, where C is concentration of EM-1, total concentration of OPRM is proportional to maximum binding capacity Rmax, Concentration of complex is measured directly as Response Unit in R. A K_D_ of 60.9±18.1 nM for EM-1 was determined by fitting the data with a 1∶1 interaction model. Error bars represent values of two duplicates.

Upon supplying increasing concentrations of agonist EM-1 to the immobilized OPRM increasing binding signal (RU) was observed (Rmax = 40 RU (EM-1: MW 610 Da)). Evaluation with a 1∶1 interaction model allowed determining a K_D_ of 61±18 nM for the binding of EM-1 to OPRM isolated in detergent FOS-12 (Figure 8), which confirmed the agonist binding capacity of the isolated OPRM.

No binding of endomorphine-1 was observed for reduced OPRM, which was immobilized on the chip in 1 mM TCEP. This negative control indicated that the endomorphine-1 binding pocket was stabilized by a disulfide-bond in OPRM.

## Discussion

Large amounts of OPRM were overproduced as inclusion bodies in RIL cells using the autoinduction method at 37°C when grown for 4 h after induction. The specific degradation into an 18 kD of N-terminal fragment of the receptor was present when expressed with in RP and RIL cells at 37°C. Possibly a flexible intracellular loop exists between the 3rd and 4th transmembrane domain, which was cleaved by a protease from *E.coli*. RP and RIL cells obviously respond to the over-expression of OPRM with cleavage of the membrane protein, whereas C41 and C43 cells direct the protein only into inclusion bodies when challenged by expression of OPRM.

The effectiveness of the two strains, C41 (DE3) and C43 (DE3), in over-expressing toxic and membrane proteins has been previously demonstrated. The strain C43 (DE3) was derived from C41 (DE3) by selecting for resistance to a different toxic protein [31]. Compared with the other E.coli strains RP and RIL, the C41 and C43 strains were observed to yield more membrane mass per cell mass. This finding may explain one of the reasons why OverExpress™ C41 (DE3) and C43 (DE3), have been found to be superior for over-expressing toxic and membrane proteins. The over-expression of OPRM was largely tolerated by C43 with the conditions of 0.4 mM IPTG at 18°C for 8–12 h. Preferential membrane-insertion of OPRM instead of formation of inclusion bodies may be due to the larger mass of membrane in these strains. The membrane insertion of the protein represented first evidence that a correctly folded and stable OPRM was obtained.

Interestingly, in contrast to the N-terminally tagged OPRM the expression of C-terminal decahis-tag OPRM in C43 gave only a poor expression level of the protein. This result appears to contradict the conclusion that a hexahistidine tag fused at the amino terminus of opioid receptor decreased expression levels markedly in baculovirus-infected insect cells [32] due to the “positive inside rule” described for *E.coli* membrane proteins and GPCRs [33]. The so-called positive inside rule states that cytoplasmic segments contain more positive charges than extra-cytoplasmic segments. This is also true for OPRM.

It was also previously claimed that due to poor expression of OPRM with a C-terminal his-tag, *E.coli* may be not a suitable expression system for OPRM [14]. In the light of our results this conclusion appears to be overly generalising.

In our case, the expression level of the N-terminally his-tagged receptor could be obtained in yields of 0.3–0.5 mg/liter of culture, which is the highest yield obtained for GPCRs from *E.coli* membrane ever reported. The obtained yield of purified OPRM is 0.17 mg/liter of culture, which corresponds to 30–60% of expressed OPRM.

Several mild detergents were used for solubilisation of the receptor, only to find solubilisation efficiency was too low and none of them was able to extract sufficient amounts of receptor except Fos-12, probably due to poor membrane breakage and solubilisation for the target protein. Further investigation of the optimal detergent e.g. Fos-14 may allow increasing the yield:expression ratio even further. The detergent Fos-14 has been reported previously to be efficient for solubilising several other GPCRs [28], [34].

The overall result improved both in yield and purity of OPRM, especially for low expression conditions, after removing the periplasmic material before cell lysis. This appears to be due to improved performance of affinity chromatography [35].

The monomeric/dimeric OPRM was separable from the aggregated state of OPRM. Thus, circular dichroism (CD) was further used to assess the state of folding of the receptor: The purified OPRM showed the predicted fraction of α-helical secondary structure as expected for a properly folded receptor, whereas the aggregated material displays reduced helicity. Anyhow, from our results it remains unclear to what extend the formation of the aggregated material with lower alpha-helicity is due to thermal or detergent induced instability of the folded protein or a principal difficulty of folding of OPRM in *E.coli*. We suppose that the membrane-integrated protein is folded. Therefore detergent induced instability appears to be the most likely cause for the appearance of a substantial fraction of protein with reduced secondary structure.

We assessed the presence of tertiary structure, respectively functionality, by the ability to bind the agonist EM-1. A K_D_ of OPRM for EM-1 (61±18 nM) was determined by Surface Plasma Resonance, which is comparable to the value published for receptor from HEK293 cells (29.9±2.9 nM) [36], if methodological differences are taken into account. Yet, agonist affinity was decreased by presumably two orders of magnitude as compared to the value measured from mammalian cells for EM-1 (360 pM) [37]. It was presumed previously that the difference between the affinity for EM-1 (29.9 nM) and that first reported value (0.36 nM) is due to the use of different receptor preparations and radio-ligands [36]. The effect of mammalian lipids could also explain the substantial difference [38].

Finally, our results on a human membrane protein, respectively GPCR, that has been previously proven to be very difficult to express, provide further evidence that a moderate expression level and a slow expression rate at low temperature should be targeted in *E.coli*. The easy scale up and speed of expression in *E.coli* compensates for the moderate yield, which is still sufficient to allow performing even crystallization experiments.

## Materials and Methods

### Materials

*E. coli* cell strains CodonPlus RP and CodonPlus RIL were purchased from Stratagene. OverExpress™ C41 (DE3) and C43 (DE3) were purchased from Lucigen. DNA encoding the human-opioid receptor was provided by Qiagen (Germany). Ni-NTA was purchased from Qiagen (Germany). Superdex 200 (16/60) and analytical grade Superdex 200 HR 10/30 size exclusion chromatography were from GE Healthcare. All other chemicals were from either Sigma-Aldrich or Fluka. Fos-12 was purchased from Anatrace (Maumee, OH) and other detergents were purchased from GLYCON (Germany). Buffer A: 20 mM Tris–HCl, 150 mM NaCl, 10% Glycerol, pH 8. Solubilisation buffer: 20 mM Tris–HCl, 300 mM NaCl, 10% Glycerol, pH 8, 1% Fos-12, 5 mM imidazole. Wash buffer: 20 mM Tris–HCl, 300 mM NaCl, 10% Glycerol, pH 8, 0.1% Fos-12, 25 mM imidazole. Elution buffer: 20 mM Tris–HCl, 300 mM NaCl, 10% Glycerol, pH 8, 0.1% Fos-12, 300 mM imidazole. Gel filtration buffer: 20 mM Tris–HCl, 150 mM NaCl, 10% Glycerol, pH 8, 0.1% Fos-12. Buffer B: 5 mM NaHPO4, 10% glycerol, 0.07% Fos-12, pH 7.5 (with or without 1 mM TCEP, as required).

### Expression of Recombinant OPRM

The synthetic human mu opioid receptor gene (GENEART) was constructed into the Qiagen plasmid pQE-2 thereby encoding full-length OPRM with either an N-terminal or C-terminal deca-histidine tag. Any codons that are rarely used in *E. coli* were avoided.

Expression with autoinduction was carried out at 37°C [39]. Plasmids were transformed into the different *E. coli* expression strains: BL21-CodonPlus-RIL and –RP, C41 (DE3) and C43 (DE3). 500 µl of an overnight preculture was used to innoculate 500 ml autoinduction media in a 2 l flask. Cultures were grown at 37°C for 5 h with shaking at 200 rpm until glucose was used up (tested by glucose test strips). Cultures were continually grown at 37°C for 5 h or overnight with shaking at 200 rpm.

Expression with IPTG at 18°C was carried out in the same *E. coli* strains. After transforming into the different strains (BL21-CodonPlus -RIL and –RP, C41(DE3) and C43 (DE3)) 2–3 fresh colonies from plates complemented with 2% glucose, were inoculated into DYT or TB containing 50 µg/ml kanamycin and 2% D-glucose at 37°C. Routinely 7 hours later, a fraction of the preculture with high cell density (typically at OD600 = 5–8) was diluted into 500 ml of the respective medium containing 50 µg/ml kanamycin to obtain a cell density at OD600 of 0.1–0.2 and grown with vigorous shaking until the OD600 reached 0.6–0.8 (usually 1 h) at 37°C. IPTG was added to a final concentration of 0.2, 0.4, 0.8 and 1 mM, and growth was continued for a further 3–5 h at 37°C or 8–12 h at 18°C.

Bacteria were then harvested by centrifugation at 6000 rpm (JLA-8.1000, Beckman) for 20 min. Pellets were flash frozen in liquid nitrogen and stored at −80°C until used for purification.

### Preparation of Inclusion Body and Membrane Fractions

All operations were carried out at 4°C or on ice. Newly harvested cells were treated with osmotic shock to remove the periplasmic fraction [35]. The pellets were resuspended in 5–10 ml/g lysis buffer. Cell lysis was achieved by using either the High Pressure Homogenizer EmulsiFlex-C3 (Avestin, Canada) or Constant Cell Disruption Systems (Constant Systems, UK) in buffer A plus 5 mM MgCl_2_, 2 mM ß-*ME*, 1 mM EDTA, DNAse, lysozyme (1 mg/ml), supplemented with EDTA-free protease inhibitors (one tablet/50–100 ml, Roche). The cell lysate was centrifuged at 1000 g to remove unbroken cell and cell debris, followed by another centrifugation at 10000 g for 40 min to collect white inclusion bodies. The supernatant was further centrifuged at 100,000 g for 1 h to harvest a membrane fraction. Pellets were flash frozen and stored at −80°C until further use.

### Detergent Screening: Small Scale Solubilisation of OPRM

1 g of the resulting membrane pellet was solubilised in 10–20 ml of solubilisation buffer (buffer A containing detergents or chaotropic agents). The following detergents were used as the solubilisers: 1% LDAO, 1% Fos-12, 1% DDM, 1% Cy6, 0.8% laurysarcosine, 1% SDS, 6 M urea. The solubilisation was allowed to proceed with gentle agitation at 4°C for 2 h. The solubilised supernatant was separated by centrifugation at 20,000 g (4°C, 0.5 h). The respective membrane fractions before and after solubilisation and the residue pellet were analyzed by western blot.

### Isolation of OPRM

The membrane pellets were solubilised at 1 g/10–20 ml of membrane/solubilisation buffer for 1–3 h at 4°C. The supernatant was separated by ultracentrifugation at 100,000 g (1 h, 4°C) and subsequently incubated with Ni-NTA resin at a ratio of 0.2–0.4 mg target protein/ml Ni-NTA resin in batch mode. The resin was then poured into a column and excess solution collected (flow-through). After washing with 10 bed volumes of wash buffer, proteins bound to the resin were eluted with 5 bed volumes with a final concentration of 300 mM imidazole in wash buffer. All the elution fractions were pooled and diluted to a final concentration of 25 mM imidazole, and then passed again onto Ni-NTA to conduct wash and elution steps as described above. As a final purification step the OPRM sample was subjected to a superdex 200 (16/60) gel filtration column (GE Healthcare) in gel filtration buffer. SDS-PAGE and western blotting were used to identify fractions containing OPRM.the samples were flash-frozen in liquid nitrogen and then stored at −20°C until further use.

### Mass Spectroscopy

To confirm the identity of the isolated protein, peptide mass fingerprints were determined by mass spectroscopy. The coomassie stained band was excised and destained. The protein was reduced with 10 mM DTT in 25 mM NH_4_HCO_3_ solution at 56°C for 1 hour and then incubated in 55 mM iodoacetamide in 25 mM NH_4_HCO_3_ for 45 min at room temperature in darkness. Gel pieces were then incubated with 20 µl trypsin (Sigma) solution (20 µg/ml) to be digested overnight at 37°C. The resulting fragments were then analyzed by a Bruker Daltonic Ultraflex III TOF/TOF mass spectrometer.

### Circular Dichroism Measurements

All CD spectra were acquired at 25°C on a Jasco J-810 spectropolarimeter using 0.1 cm path length cylindrical quartz cuvettes. The purified protein was desalted by a PD10 (GE Healthcare) column and subsequently concentrated by ultrafiltration (Amicon, Millipore). Measurements were performed in Buffer B to reduce background signal. Protein concentrations were determined by UV-spectrometry using extinction coefficients [40]. The CD spectra were obtained from 190 to 280 nm using a bandwidth of 1 nm, a step width of 0.5 nm, a scanning speed of 100 nm/min and a time constant of 0.5 s. For data processing background correction and data accumulation (20 spectra) were used. Data were converted into units of mean residue ellipticity (deg cm^2^ dmol^−1^) and analyzed for secondary structure with the program K2D [41].

### Ligand Binding Assays by Surface Plasmon Resonance

The binding experiments were carried out on a Biacore-X instrument (Biacore) at 25°C. OPRM was immobilized in one cell within a Ni-NTA sensor chip to obtain around 4000 response units (RU). The second cell was used as a control. Both cells were equilibrated with running Buffer B to establish a stable baseline. EM-1 was dissolved in buffer B and injected (flow rate 5 µl/min) over the captured receptor and the reference cell at concentrations of 10, 30, 50, 60, 80, and 100 nM. Association was monitored for 2 min, and dissociation was monitored for 5 min. No regeneration was performed between EM-1 injections. Data analysis was carried out by using BIAevaluation software using an 1∶1 interaction model.
